# Predictive and prognostic value of aurora kinase A combined with tumor-infiltrating lymphocytes in medullary thyroid carcinoma

**DOI:** 10.3389/fonc.2024.1379420

**Published:** 2024-06-06

**Authors:** Zhongyu Wang, Fengli Guo, Guiming Fu, Zewei Zhao, Ning Kang, Xiukun Hou, Xiangqian Zheng

**Affiliations:** ^1^ Department of Thyroid and Neck Cancer, Tianjin Medical University Cancer Institute and Hospital, National Clinical Research Center for Cancer, Key Laboratory of Cancer Prevention and Therapy, Tianjin’s Clinical Research Center for Cancer, Tianjin, China; ^2^ Department of Thyroid and Breast Surgery, Binzhou Medical University Hospital, Binzhou, China; ^3^ Thyroid-otolaryngology Department, Sichuan Clinical Research Center for Cancer, Sichuan Cancer Hospital & Institute, Sichuan Cancer Center, Affiliated Cancer Hospital of University of Electronic Science and Technology of China, Chengdu, China

**Keywords:** aurora kinase A, tumor-infiltrating lymphocyte, medullary thyroid carcinoma, structural recurrence, biochemical recurrence

## Abstract

**Background:**

Aurora kinase A (AURKA) and tumor-infiltrating lymphocytes (TILs) are both known to play an essential role in tumorigenesis. However, the expression and prognostic value of the AURKA and TILs in medullary thyroid carcinoma (MTC) have not yet been investigated.

**Patients and methods:**

Surgical specimens and clinical data of 137 patients diagnosed with MTC were collected. AURKA expression and TILs infiltration were quantified by immunohistochemistry and hematoxylin-eosin staining. Subsequently, the prognostic value of AURKA expression and TIL infiltration in MTC was evaluated.

**Results:**

AURKA was highly expressed in patients with multifocal tumor, cervical lymph node metastasis, and an advanced TNM stage, indicating a high probability of recurrence. AURKA further exhibited a positive correlation with TILs (R = 0.44, *P* < 0.001). High expression of AURKA combined with a low numbers of TILs (AURKA^high^/TILs^low^) was identified as an independent prognostic factor for biochemical recurrence (odds ratio: 4.57, 95% confidence interval: 1.54–14.66, *P* < 0.01) and recurrence-free survival (hazard ratio: 3.64, 95% confidence interval: 1.52–8.71, *P* < 0.001). The combination of AURKA and TILs apparently improves the prognostic value for biochemical recurrence (area under the curve: 0.751) and structural recurrence (area under the curve: 0.836) of MTC. Notably, AURKA^high^/TILs^low^ demonstrated a high value for prediction of distant or unresectable locoregional recurrence, with an overall accuracy of 86.9%.

**Conclusion:**

AURKA^high^ is associated with the MTC malignancy. The combination of AURKA^high^/TILs^low^ was identified as novel independent prognostic marker in MTC, predicting incurable disease recurrence with high accuracy.

## Introduction

Medullary thyroid carcinoma (MTC) is a form of neuroendocrine tumors originating from calcitonin‐producing parafollicular cells (C cells). These tumors account for 5%–10% of all thyroid malignancies (1). Approximately 75% of cases are sporadic, but MTC accounts for 14% of all thyroid cancer-related deaths. MTC is prone to cervical lymph node metastasis in the early stages of the disease, and distant metastasis is detected in 10% of patients with MTC at initial diagnosis. If not treated in a timely manner, the condition will rapidly deteriorate. Surgery in combination with adjunctive therapy (e.g., radiotherapy and chemotherapy) is an effective treatment option for local MTC (1, 2). In recent years, research has identified various markers which display prognostic value in MTC. Among them, calcitonin and carcinoembryonic antigen (CEA) are used to monitor the progression of MTC. Further, it is currently thought that, following the diagnosis of MTC, a steady rise in calcitonin and CEA levels following the diagnosis of MTC is indicative of poor prognosis. However, a small proportion of healthy individuals exhibit some increase in calcitonin levels, while the CEA values in patients with MTC are mostly within the normal range. Therefore, there are certain limitations in the use of these marked for disease monitoring (3). Although numerous molecular markers have been proposed, at present, there are no absolutely effective biomarkers that can predict the prognosis of MTC.

Aurora kinase A (AURKA) is an important member of the aurora kinase family, which are involved in the regulation of cell mitosis and progression through the cell cycle (4). AURKA is primarily responsible for the replication and separation of the centrosome, the aggregation of the bipolar spindle apparatus, and the entry and exit of mitosis. In addition, AURKA plays an important role in the maturation of the centrosome and the assembly of the spindle apparatus (5). Owing to the important biological functions of AURKA, phase 2 clinical trials have been launched in recent years for the to develop targeted inhibitors (6, 7). Studies have demonstrated that the expression of AURKA is upregulated in breast cancer, prostate cancer, ovarian cancer, pancreatic cancer, and other types of tumors (8). This upregulation leads to malignant proliferation, epithelial–mesenchymal transformation, migration, and invasion of tumors cells, and has been associated with poor patient prognosis (9–11). Previous studies have further suggested that high expression of AURKA (AURKA^high^) in breast cancer is a significant adverse prognostic factor for overall survival (OS). Moreover, AURKA is closely related to disease recurrence in obese patients with early-stage breast cancer (12), while AURKA^high^ in patients with gastric cancer has been linked to a higher risk of death (13). Given this context, the present this study aimed to investigate the potential predictive value of AURKA in MTC.

Tumor-infiltrating lymphocytes (TILs) include T and B cells that can recognize infiltrating high-risk cancer cells and promote their apoptosis. Many studies have found that TILs play a biphasic role in the occurrence and development of tumors, alternatively participating in antitumor immune responses to inhibit extension of tumor, while also functioning to mediate the production of cytokines and other molecules to form an immunosuppressive microenvironment, resulting in immune escape, and driving tumor recurrence (14–16). Furthermore, research has shown that TIL number has potential as a prognostic factor for tumor cell survival. In fact, numerous studies have reported that TILs are important prognostic factors in several types of cancer, including lung, liver, esophageal, gastric, colorectal, low-grade glioma, female breast, and cervical cancers (17–24). Furthermore, studies on colorectal cancer have shown that the individual immune cell subtype is not a good predictor for cancer prognosis (25). TIL infiltration is a coordinated and interactive process, and research has shown that the overall TIL number is predictive of OS and disease free survival (DFS) of colorectal cancer (25). Recent research has further demonstrated that patients with early-stage triple-negative breast cancer and high level of TILs have significantly lower rates of distant recurrence and longer recurrence-free survival (RFS) (26). In addition, a study of the relationship between tumor deposits and TILs in gastric cancer concluded that their interaction influenced both DFS and OS (27). MTC is an immunologically active tumor with a higher organized immune infiltration than expected (28), rendering TILs a strong possibility as favorable tumor predictors.

It is well known that immunogenic cancer cells cause immune escape by modulating the inactivation of initially recruited immune components (29). In a mouse model of breast cancer, treatment with the Aurora-A inhibitor alisertib (MLN8237) caused intensive cancer cell apoptosis, resulting in a reduction in myeloid-derived suppressor cells and tumor-associated macrophages (30). This Aurora-A inhibitor disrupted the immunosuppressive environment, without harming T cells. Further, another study showed that treatment with Aurora-A inhibitors or loss of the AURKA gene could increase the number of CD8+T cells in tumors, thus improving the prognosis of patients with colorectal cancer (31). Therefore, we speculate that AURKA is related to immune cells, and can play a role in promoting cancer by affecting the number of immune cells to a certain extent.

In this study, we evaluated AURKA expression and TIL infiltration in MTC by immunohistochemistry (IHC) and hematoxylin-eosin staining(HE), and explored their associations with clinicopathological characteristics. We hypothesized that AURKA and TILs would jointly affect the prognosis of MTC, and therefore further evaluated the predictive value of combined AURKA and TILs in patients with disease recurrence.

## Patients and methods

### Patient selection and tissue samples

We recruited patients with MTC treated in from Tianjin Medical University Cancer Institute and Hospital (Tianjin, China) from 2011 to 2017. The inclusion criteria were: 1) no history of previous neoadjuvant therapy, including radiotherapy, chemotherapy, endocrine therapy, target therapy, or immunotherapy; 2) absence of any other malignant tumors; 3) undergoing primary operation; 4) complete-case data; and 5) availability of paraffin-embedded sections of thyroid tumor tissue and adjacent normal tissue from Tianjin Medical University Cancer Institute and Hospital. Clinical staging was based on the 8th edition of the American Joint Committee on Cancer (AJCC) staging system. All patients provided written informed consent for their participation. Demographics, clinical characteristics and outcomes, pathological results, and biochemical examinations of all patients were obtained with their permission from the patients. All studies on patients were performed following ethics committee approval.

### Histology and IHC

Paraffin-embedded tissue samples obtained from patients with MTC were sectioned, and soaked in citrate buffer for 8 min in a pressure cooker to repair antigen. The samples were then treated with hydrogen peroxide to block peroxidase activity, sealed with 5% bovine serum albumin, and incubated with rabbit anti-human AURKA primary antibody (Abcam ab52973; 200 μL; antibody concentration 1:200) overnight. After washing with phosphate-buffered saline, the slides were incubated with secondary antibodies under normothermic conditions for 2 h. Pathological specimens were collected and reviewed by three highly experienced pathologists.

**Figure 1:**
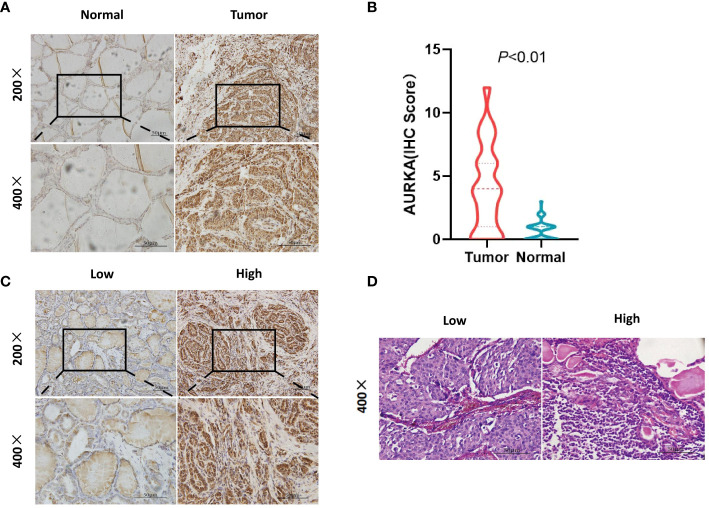
Immunohistochemistry (IHC) staining of aurora kinase A (AURKA) and hematoxylin-eosin staining of tumor-infiltrating lymphocytes (TILs). **(A, B)** AURKA positivity in the nucleus or cytoplasm of cells. The expression was higher in the tumor versus surrounding normal tissues (P<0.01). **(C)** According to the scoring standard, samples were divided into two groups based on high (4–12) and low (0–3) AURKA scores. **(D)** Different numbers of TILs were divided into two groups, namely low (<40%) and high (≥40%).

The expression of AURKA and presence of peritumoral TILs was evaluated based on the following criteria. The immunohistochemical (IHC) score of AURKA was assessed based on the staining intensity (0, negative; 1, weak; 2, mild; 3, strong) and staining ratio (0%, 0; 1%–20%, 1; 21%–50%, 2; 51%–70%, 3; 81%–100%, 4). The final IHC score was calculated as: staining intensity × staining ratio. Patient samples were classified into two groups: high (4–12) and low (0–3) based on the AURKA scores (**Figure 1C**). Three pathologists performed tissue embedding sections, and independently evaluated the scores using a microscope under 10× and 20× lenses. They were unaware of the clinical follow-up data. If 2/3 experts agreed with the rating results, then the score was determined. If there were significant differences, experts reassessed the results until a consensus could be reached. The TIL score was calculated as the percentage of tumor stroma containing infiltrating lymphocytes (area occupied by mononuclear cells in tumor stroma/total stromal area). Similarly, the TIL staining scores were divided into two groups: low (<40%) and high (≥40%) (**Figure 1D**). Three pathologists performed tissue embedding sections, and independently evaluated the slides using a microscope under 40× lens. They were unaware of the clinical follow-up data. If 2/3 experts agreed with the rating results, then the score was determined. If there were significant differences, experts reassessed the results until reaching a consensus.

### Follow-up information

The follow-up methods of the study included telephone follow-up or checking of patient visit records through the electronic medical record system. Follow-up was conducted from the first day of surgery until death occurred. The median follow-up time was 81.4 (74.97–87.87) months, and the patients were followed up every 3–4 months after surgery. Structural recurrence (STR) was defined as the development of cervical, mediastinum lymph node, and distant organ metastases >6 months after surgery (confirmed by radiological imaging and pathology). RFS was calculated from the date of MTC diagnosis to that of structural recurrence or last follow-up, excluding biochemical recurrence. The censoring date for the follow-up data was March 30, 2023. Reference to the American Thyroid Association guidelines and related literature defines biochemical therapy as postoperative calcitonin levels within the reference range. Herein, biochemical recurrence (BCR) was defined as consistently elevated postoperative serum levels of calcitonin above the corresponding reference range during the study period (32–34). Overall survival (OS) was defined as the survival from the end of surgery until death from cancer.

### Statistical analysis

All statistical analyses were performed and charts were produced using the R programming language (Version 4.2.2),SPSS(26.0) and Prism (8.0) software. The association of AURKA expression with clinicopathological variables was evaluated using the chi-squared or Fisher’s exact test. The inter-group difference in AURKA expression between groups was examined with the Wilcoxon test. Univariate and multivariate logistic regression analyses for BCR were performed to generate odds ratios (OR) and 95% confidence intervals (95%CI) of factors. Moreover, univariate and multivariate Cox regression analyses for structural RFS were conducted to generate hazard ratios (HR) and the 95%CI of factors. Receiver operating characteristic curves (ROC) were drawn to quantify the predictive ability of each biomarker. Survival curves were plotted using the Kaplan–Meier method. Statistical analysis was performed, and charts were produced using the R programming language (Version 4.2.2) and the Prism (8.0). All analyses were two-sided, and P-values <0.05 indicate statistically significant differences.

## Results

### Patient characteristics

A total of 137 patients with MTC were included, with a median follow-up time of 82.63 months. The median age of the patients was 50 years (range: 14–76 years); the proportions of females (N = 72, 51.1%) and males (N = 65, 48.9%) were similar in this study. According to the 8th edition of the AJCC staging system, 38 (27.7%) patients were classified as stage I–II and 99 (72.3%) as stage III–IV. All patients underwent surgical treatment. 75 patients (57.4%) experienced BCR, of which 24 patients suffered STR. 32 patients (23.4%) suffered from STR, of which 24 patients experienced BCR. 68 patients had a biochemical cure after surgery, but 48 of them eventually had a BCR one year later. In the end, 20 patients (14.6%) had a complete response, and 10 patients (7.3%) expired due to MTC until the last follow-up Details of the baseline characteristics are shown in **Table 1**.

**Table 1:** Patient clinicopathologic characteristics and events during the study period.

Characteristic	Overall cohort (N = 137)	AURKA^low^ (N = 64)	AURKA ^high^ (N = 73)	*P*-value
**Age**				0.784
<50 years	70 (51.1%)	34 (48.6%)	36 (51.4%)	
≥50 years	67 (48.9%)	30 (44.8%)	37 (55.2%)	
**Sex**				0.523
Male	65 (47.4%)	28 (43.1%)	37 (56.9%)	
Female	72 (52.6%)	36 (50.0%)	36 (50.0%)	
**Concomitant HT**				0.188
No	84 (61.3%)	35 (41.7%)	49 (58.3%)	
Yes	53 (38.7%)	29 (54.7%)	24 (45.3%)	
**Multifocal tumor**				**0.030**
No	98 (71.5%)	52 (53.1%)	46 (46.9%)	
Yes	39 (28.5%)	12 (30.8%)	27 (69.2%)	
**Bilateral tumors**				0.645
No	119 (86.9%)	57 (47.9%)	62 (52.1%)	
Yes	18 (13.1%)	7 (38.9%)	11 (61.1%)	
**Size**				0.282
<2 cm	65 (47.4%)	34 (52.3%)	21 (32.3%)	
≥2cm	72 (52.6%)	30 (41.7%)	42 (58.3%)	
**N stage**				**0.004**
N0	43 (31.4%)	24 (55.8%)	19 (44.2%)	
N1a	23 (16.8%)	15 (65.2%)	8 (34.8%)	
N1b	71 (51.8%)	25 (35.2%)	46 (64.8%)	
**Stage**				0.069
I–II	38 (27.7%)	23 (60.5%)	15 (39.5%)	
III–IV	99 (72.3%)	41 (41.4%)	58 (58.6%)	
TILs				
Low	67 (48.9%)	39 (58.2%)	28 (41.8%)	**0.014**
High	70 (51.1%)	25 (35.7%)	45 (64.3%)	
Events				
Structural recurrence	32 (23.4%)	10 (31.3%)	22 (68.8%)	0.072
Biochemical recurrence	75 (54.7%)	27 (36.0%)	48 (64.0%)	**0.010**
MTC-related death	10 (7.3%)	3 (30.0%)	7 (70.0%)	0.441

AURKA, aurora kinase A; AURKA^high^, high expression of AURKA; AURKA^low^, low expression of AURKA; HT, Hashimoto’s thyroiditis; MTC, medullary thyroid carcinoma; TILs, tumor-infiltrating lymphocytes. Bold values means statistically significant (P<0.05).

### Clinicopathological associations and immunohistochemical findings

We assessed AURKA expression and TIL infiltration in MTC tumor tissues using IHC staining and HE staining. AURKA positivity in the nucleus or cytoplasm was confirmed (**Figure 1A**); its expression in tumor cells was validated as being higher in surrounding cancerous tissues than normal tissues (**Figure 1B**). AURKA expression was predominantly detected in tumor tissues, and approximately 107 patients (78.1%) stained positive; of which, 28, 49, and 30 samples had weak, mild, and strong staining, respectively. In total, 73 and 64 patients had AURKA^high^ and AURKA^low^ expression, respectively (**Figure 1C**). The median percentage of lymphocyte infiltration in tumor stroma was 40%. Tumors with AURKA^high^ tended to be characterized by more extensive infiltration of TILs than those with low AURKA expression (*P* = 0.014) (**Figure 1D**). Representative images of AURKA expression and TILs are displayed in **Figure 1**.

**Figure 2:**
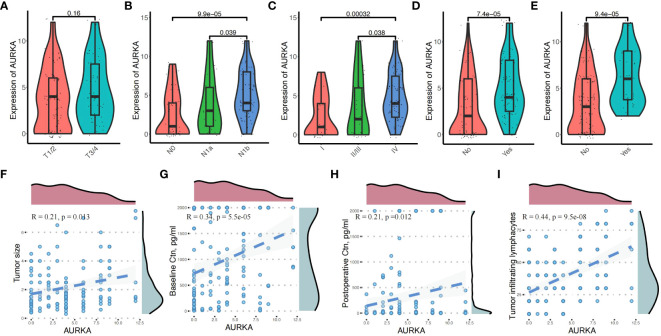
Correlation between aurora kinase A (AURKA) expression and the clinicopathologic characteristics in patients with medullary thyroid carcinoma. **(A)** There was no difference in AURKA expression between patients with T1/2 and T3/4 stage disease. **(B, C)** Significant differences in N1b (*P* < 0.05) and more advanced TNM stage (*P* < 0.01). **(D, E)** Patients with biochemical recurrence (BCR) and structural recurrence (STR) have higher IHC scores. **(F–I)** High expression of AURKA was correlated with tumor size (R = 0.21, P = 0.013), preoperative (R = 0.34, *P* < 0.001) and postoperative basal serum calcitonin (R = 0.21, *P* = 0.012), and tumor-infiltrating lymphocytes (TILs) (R = 0.44, *P* < 0.001).

Chi-squared analysis revealed that the expression levels of AURKA were closely related to multifocal tumor (P = 0.030), N stage (P = 0.004), TILs (P = 0.014), and BCR (P = 0.010) (**Table 1**). There was no difference in AURKA expression between patients with T1/2 stage and T3/4 stage disease (P = 0.160) (**Figure 2A**). However, we found that AURKA expression was increased in tumors with a higher degree of malignancy, particularly in those with N1b (P < 0.001) or advanced TNM stage (P < 0.001) (**Figures 2B, C**). Further, the patients who developed STR (*P* < 0.001) (**Figure 2D**) or BCR (*P* < 0.001) (**Figure 2E**) tended to be associated with high AURKA IHC scores. These results indicated that AURKA may be closely related to the invasion and metastasis of tumors, and may have resulted in a more malignant phenotype of MTC. In addition, AURKA expression was correlated with tumor size (R = 0.21, P = 0.013), preoperative (R = 0.34, *P* < 0.001) and postoperative basal serum levels of calcitonin (R = 0.21, *P* = 0.012), and TILs (R = 0.44, *P* < 0.001) (**Figures 2F–I**).

### Prognostic value of AURKA combined with TILs for MTC

**Figure 3:**
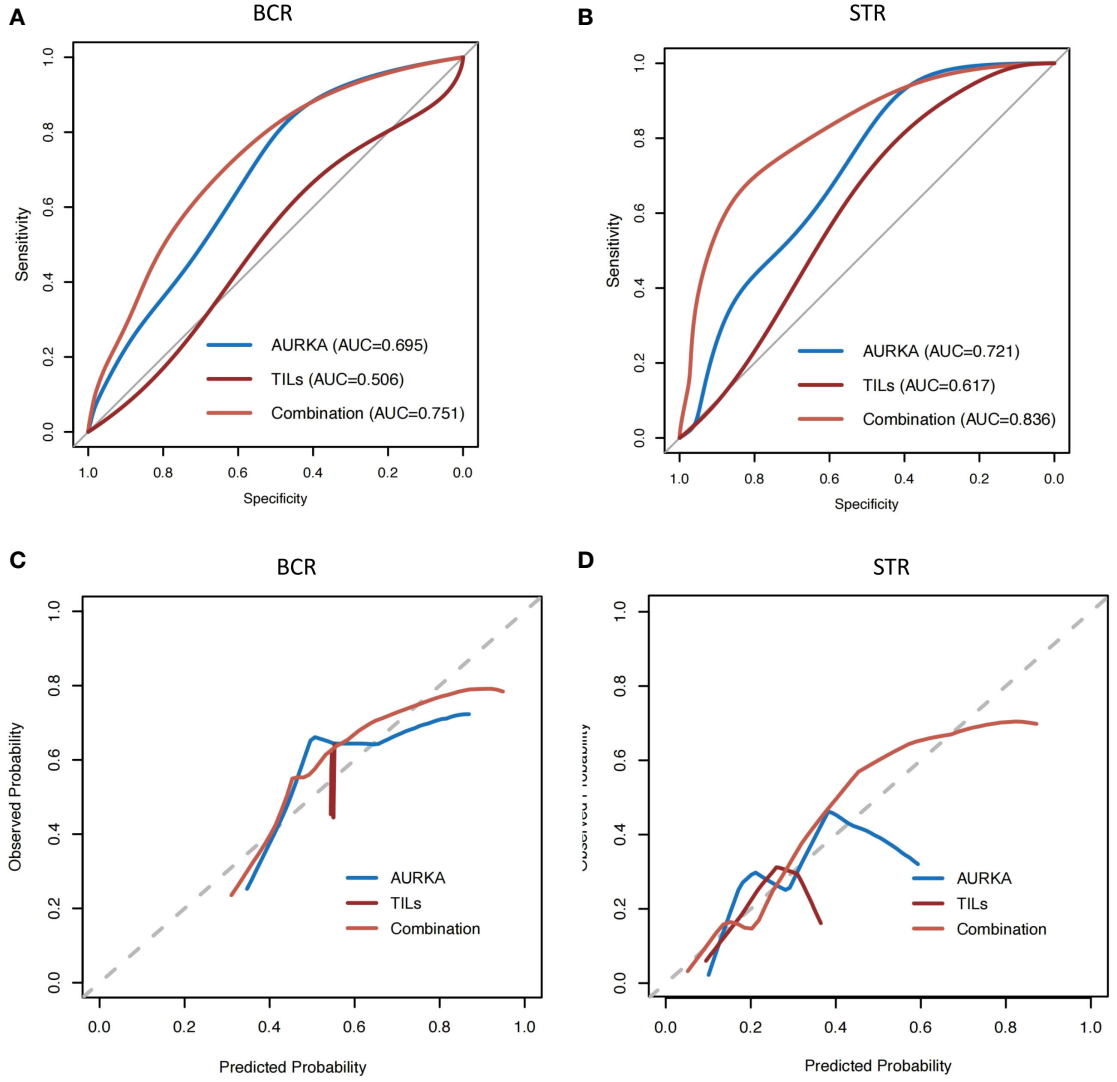
The AUC value of AURKA combined with TILs was higher than those analyzed separately. **(A, B)** Receiver operating characteristic curve analysis of aurora kinase A (AURKA) expression and tumor-infiltrating lymphocytes (TILs), and combination analysis. **(C, D)** Calibration plots for biochemical recurrence (BCR) and structural recurrence (STR). AUC, area under the curve of receiver operating characteristic curve.

Because AURKA was associated with STR and BCR, we conducted receiver operating characteristic curve analysis to quantify the predictive ability of AURKA and TILs. The aim was to distinguish patients with STR or BCR from those exhibiting complete response. The ability of AURKA to predict STR and BCR was found to be limited, with an area under the curve (AUC) of 0.721 and 0.695 respectively. We subsequently evaluated the predictive value of TILs, which are considered an essential biomarker in multiple types of solid cancer. Similarly, the AUC values of TILs were 0.617 and 0.506 for STR and BCR, respectively, showing low value in terms of clinical application. Finally, we constructed a new model that combined AURKA expression and TILs by way of AURKA/TILs. After combination, the AUC value reached 0.836 and 0.751 for STR and BCR, respectively (**Figures 3A–D**); notably, these values were markedly higher for either of the factors alone.

**Figure 4:**
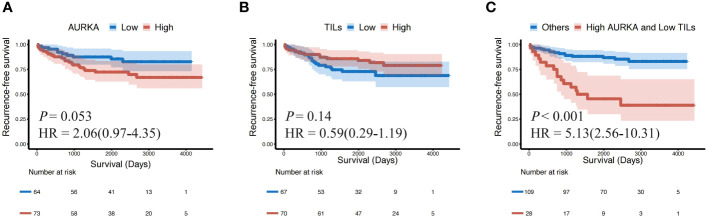
The patients with AURKA^high^ and TILs^low^ showed a shorter recurrence-free survival (RFS) than others. Kaplan–Meier survival plots presenting recurrence-free survival (RFS) of the **(A)** AURKA^low^ and AURKA^high^ groups; **(B)** TILs^low^ and TILs^high^ groups; and **(C)** AURKA^high^ and TILs^low^, and other groups. AURKA, aurora kinase A; AURKA^high^, high expression of AURKA; AURKA^low^, low expression of AURKA; HR, hazard ratio; TILs, tumor-infiltrating lymphocytes; TILs^high^, high level of TILs; TILs^low^, low level of TILs.

The Kaplan–Meier survival analysis revealed that patients with AURK^high^ had shorter RFS than those with lower AURKA expression (*P* = 0.053, HR: 2.06, 95%CI: 0.97–4.35), while high numbers of TILs were related to longer RFS than low numbers (*P* = 0.14, HR: 0.59, 95%CI: 0.29–1.16), however this did not reach achieve statistically significant (**Figures 4A, B**). When we combined AURKA and TILs, patients with AURKA^high^ and low numbers of TILs (TILs^low^) demonstrated a significantly shorter RFS than other patients with neither or only one of these features (*P* < 0.001, HR: 5.13, 95%CI: 2.56–10.31) (**Figure 4C**).

### AURKA^high^ combined with TILs^low^ (AURKA^high^/TILs^low^) independently predicted BCR and RFS

In the univariate analysis, tumor size ≥2 cm, metastatic lymph nodes ≥6, metastatic lymph node ratio ≥0.2 and AURKA^high^/TILs^low^ were associated with an increased risk of BCR in patients with MTC. However, only the metastatic lymph node ratio ≥0.2 (OR: 3.41, 95%CI: 1.08–10.72, P = 0.04) and AURKA^high^/TILs^low^ (OR: 3.97, 95%CI: 1.92–8.19, *P* < 0.01) were identified as independent predictors of BCR in the multivariate analysis (**Table 2**).The results of the univariate Cox regression analysis revealed that multifocal tumors, tumor size ≥2 cm, ≥6 metastatic lymph nodes, metastatic lymph node ratio ≥0.2, AURKA^high^ and AURKA^high^/TILs^low^ were associated with RFS. The multivariate analysis further indicated that a metastatic lymph node ratio ≥0.2 (HR: 4.27, 95%CI: 1.24–16.00, *P* = 0.02) and AURKA^high^/TILs^low^ (HR: 8.82, 95%CI: 2.62–33.80, *P* < 0.01) were independent prognostic factors of RFS in patients with MTC (**Table 3**).

**Table 2:** Univariate and multivariate logistic analyses for biochemical recurrence in patients with MTC.

Characteristic	Univariate		Multivariate	
	OR (95%CI)	*P*-value	OR (95%CI)	*P*-value
Age				
<50 years				
≥50 years	0.82 (0.41–1.65)	0.57		
Sex				
Male				
Female	0.81 (0.40–1.62)	0.55		
Concomitant HT				
No				
Yes	1.43 (0.71–2.90)	0.32		
Multifocal				
No				
Yes	0.94 (0.44–2.04)	0.88		
Bilateral tumors				
No				
Yes	0.95 (0.33–2.70)	0.92		
Size				
<2 cm				
≥2 cm	2.25 (1.06–4.75)	0.03	1.40 (0.64–3.04)	0.40
Metastatic lymph nodes				
<6				
≥6	2.93 (1.36–6.35)	<0.01	0.79 (0.25–2.42)	0.67
Lymph node ratio				
<0.2				
≥0.2	4.45 (1.92–10.29)	<0.01	3.41 (1.08–10.72)	**0.04**
<10				
≥10	2.87 (1.33–6.21)	<0.01	1.68 (0.71–3.95)	0.24
TILs				
Low				
High	0.59 (0.29–1.19)	0.14		
AURKA expression				
Low				
High	2.06 (0.97–4.35)	0.06		
Combination				
Others				
AURKA^high^ and TILs^low^	5.13 (2.56–10.31)	<0.01	3.97 (1.92–8.19)	**<0.01**

95%CI, 95% confidence interval; AURKA, aurora kinase A; AURKAhigh, high expression of AURKA; OR, odds ratio; HT, Hashimoto’s thyroiditis; MTC, medullary thyroid carcinoma; TILs, tumor-infiltrating lymphocytes; TILslow, low level of TILs. Bold values means statistically significant (P<0.05).

**Table 3:** Univariate and multivariate Cox analyses for structural recurrence-free survival in patients with MTC.

Characteristic	Univariate		Multivariate	
	HR (95%CI)	*P*-value	HR (95%CI)	*P*-value
Age				
<50 years				
≥50 years	0.73 (0.37–1.43)	0.36		
Sex				
Male				
Female	0.52 (0.26–1.04)	0.06		
Concomitant HT				
No				
Yes	0.78 (0.39–1.55)	0.48		
Multifocal				
No				
Yes	2.34 (1.07–5.15)	**0.03**	1.13 (0.38–3.26)	0.82
Bilateral tumors				
No				
Yes	2.39 (0.8–7.13)	0.12		
Size				
<2 cm				
≥2 cm	3.2 (1.59–6.44)	**<0.01**	1.49 (0.57–4.00)	0.42
Positive lymph nodes				
<6				
≥6	4.61 (2.23–9.52)	**<0.01**	1.11 (0.33–3.73)	0.87
Lymph node ratio				
<0.2				
≥0.2	8.26 (3.8–17.94)	**<0.01**	4.27 (1.24–16.00)	**0.02**
TILs				
Low				
High	0.96 (0.49–1.89)	0.91		
AURKA expression				
Low				
High	2.63 (1.32–5.26)	**0.01**	0.58 (0.16–1.87)	0.37
Combination				
Others				
AURKA^high^ and TILs^low^	3.87 (1.46–10.3)	**0.01**	8.82 (2.62–33.80)	**<0.01**

95%CI, 95% confidence interval; AURKA, aurora kinase A; AURKA^high^, high expression of AURKA; HT, Hashimoto’s thyroiditis; HR, hazards ratio; MTC, medullary thyroid carcinoma; TILs, tumor-infiltrating lymphocytes; TILslow, low level of TILs. Bold values means statistically significant (P<0.05).

### AURKA^high^/TILs^low^ indicated a higher risk of distant or unresectable locoregional recurrence

**Figure 5:**
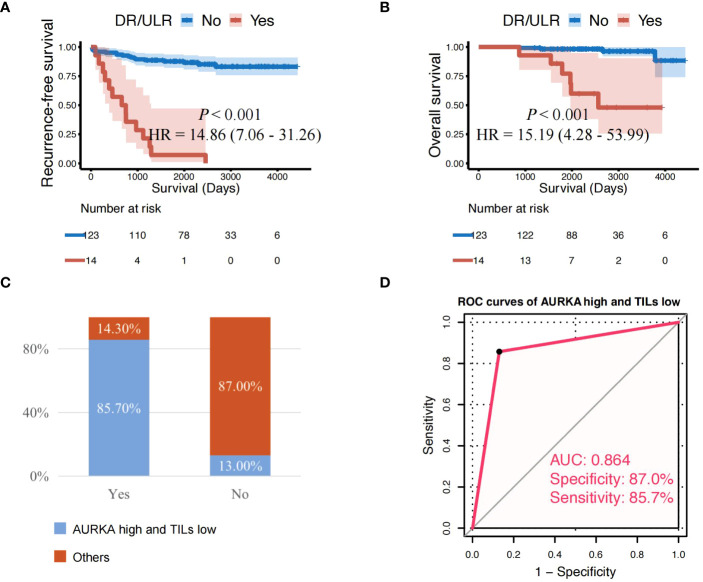
Patients with AURKA ^high^/TILs ^low^ have a higher risk of distant or unresectable local recurrence. **(A, B)** Cases with distant recurrence (DR) or unresectable locoregional recurrence (ULR) showing significantly shorter recurrence-free survival and overall survival than those without. **(C)** Proportion of AURKA^high^ and TILs^low^ with developed DR or ULR among all patients. **(D)** AURKA^high^ combined with TILs^low^ demonstrated high predictive value in patients with DR or ULR (overall accuracy: 86.9% [119/137]; and AUC: 0.864). AUC, area under the curve; AURKA, aurora kinase A; AURKA^high^, high expression of AURKA; AURKA^low^, low expression of AURKA; HR, hazard ratio; TILs, tumor-infiltrating lymphocytes; TILs^low^, low level of TILs.

Patients who developed distant or unresectable locoregional recurrence during the surveillance period were often treated with palliative care. As a result, those patients demonstrated extremely poor treatment effect and low survival rates [26]. In this study, patients with distant recurrence (DR) or unresectable locoregional recurrence (ULR) had a significantly shorter RFS (HR: 14.86, 95%CI: 7.06–31.26, *P* < 0.001) and OS (HR: 15.19, 95%CI: 4.28–53.99, *P* < 0.001) than those without (**Figures 5A, B**). Interestingly, we also found that the majority of patients (85.7%) who developed distant or unresectable locoregional recurrence (ULR) were characterized by both AURKA^high^ and TILs^low^ (**Figure 5C**). AURKA^high^/TILs^low^ demonstrated high predictive value in patients with distant recurrence or unresectable locoregional recurrence, with an overall accuracy of 86.9% (119/137) and AUC of 0.864 (**Figure 5D**).

## Discussion

In this study, we established an independent cohort by randomly selecting 137 patients with MTC to investigate the predictive and prognostic value of AURKA and TILs. The expression of AURKA was associated with a high degree of MTC malignancy, as well as cervical lymph node metastasis and recurrence. We further found that the combination of AURKA with TILs exhibited significantly improved prognostic value compared with AURKA only or TILs alone in MTC. In addition, AURKA^high^/TILs^low^ was identified as an independent prognostic factor of both BCR and STR, allowing the accurate prediction of incurable disease recurrence in patients with MTC. These results are clinically significant in terms of the adoption of individualized treatment for patients with different prognoses.

Although AURKA has been extensively investigated as a carcinogen in numerous human malignancies as a carcinogen, its prognostic value in MTC remains controversial. A pooled analysis of the AURKA mRNA levels of patients with MTC did not reveal any statistically significant alterations in AURKA expression across tumors different TNM stages. However, one study notably showed that the use of AURKA inhibitors could prevent the proliferation and tumorigenicity of MTC *in vitro* (35). However, another study found that AURKA expression was associated with advanced TNM stage and regional lymph node metastasis in a small cohort of 71 patients with MTC (36). As such, there is an urgent need to investigate the predictive value of AURKA for the prognosis of MTC. Given this context, in the present study, we analyzed the expression levels of AURKA in 137 patients. AURKA was not found to be associated with the T stage, but was highly expressed in patients with multifocal tumor and advanced tumor stage. Particularly, we found that AURKA^high^ was linked to regional lymph node metastasis and disease recurrence, suggesting that AURKA is associated with MTC aggressiveness. However, AURKA was not a significant predictive factor of RFS. Higher expression of AURKA also has limited predictive ability for RFS than lower AURKA expression (P = 0.053, HR: 2.06, 95%CI: 0.97–4.35). Nevertheless, these previous studies were characterized by limitations, namely the small sample size (only 26 patients) and measurement of only mRNA levels. Thus, we think that the present findings and conclusions are reliable because of the larger sample size included in this investigation and the measurement of quantification protein levels. There are several steps at which cells can undergo changes, including mRNA translation initiation, elongation, and post-translational modification to form a protein with a specific structure and function; all these changes can result in alterations at the protein levels. Therefore, the measurement of protein levels is more reliable than that of mRNA levels.

TILs acts as a “double-edged sword” in tumor progression, as different subsets of TILs play distinct and opposing roles in various cancers. Previous studies have proposed that TILs affect lymph node metastasis and UICC stage of urinary bladder cancer, while low TILs is predictive of poor prognosis in patients with urinary bladder cancer (37). Additionally, increased numbers of TILs in the stroma before or after therapy are typically linked to a better response to therapy, and have superior predictive value for long-term prognosis in breast cancer (26, 38–40). Considering the complex pathogenesis of thyroid cancer, its recurrence is closely related to the immune microenvironment. Further discovery of the role of useful tumor markers combined with the immune microenvironment in cancer development is expected to improve on current treatment strategies for thyroid cancer.

Thus far, it has been well-established that the infiltration of immune cells affects the recurrence of thyroid cancer (41). One study which investigated 38 thyroid cancer surgical sections, including 35 cases of papillary carcinoma and 3 of myeloid carcinoma, found that the sections of thyroid cancer patients without recurrence showed varying degrees of TILs. TILs were not observed in surgical sections of patients with recurrence (42). These results indicate that TILs may be closely related to the recurrence of thyroid cancer, which needs to be further explored in more experiments in MTC. However, thus far, only a few research studies have investigated the predictive role of TILs in MTC. In one retrospective study of 95 patients with MTC, statistical investigation of RFS and OS showed that patients lacking lymphocytic infiltration were ultimately more likely to have tumor recurrence and death (43). In our study, we also explored the relationship between TILs and relapse by examining the presence of TILs in tumor tissues obtained from 137 patients with MTC Our results showed that the ability of lower numbers of TILs alone to predict RFS may be insufficient (P = 0.14, HR: 0.59, 95%CI: 0.29–1.16). as was the predictive value of AURKA expression alone. Remarkably, patients with both AURKA^high^ and TILs^low^ were associated with an obviously shorter RFS than others. Furthermore, the AURKA^high^/TILs^low^ phenotype exhibited a strong ability to accurately predict incurable disease recurrence with high sensitivity and specificity (i.e., 85.7% and 87.0%, respectively). We thus believe that AURKA^high^/TILs^low^ may be a prognostic biomarker that can accurately identify patients at high risk of incurable disease recurrence and poor survival outcomes. Therefore, we hypothesized that investigation of AURKA expression and TILs quantification may serve as a predictor to guide future treatment.

In one prior study investigating a tumor marker and TILs in MTC, the authors suggested that this tumor marker (HHLA2) and TILs are closely related, which together affect the prognosis and survival of MTC (44). Similar to this study, we also explored tumor markers and TILs, but the difference is that we analyzed the overall TILs instead of a specific subtype. Because tumor lymphocyte infiltration involves a coordinated infiltration of multiple inflammatory cells, they cannot be restricted solely to observing individual subtypes. This view has been confirmed in colorectal and breast cancer, and even regulated in breast cancer (25, 45). Moreover, in this study, we found that MTC tumors with AURKA^high^ were characterized by extensive TIL infiltration. However, AURKA^high^ (HR: 2.06, 95%CI: 0.97–4.35) and high TIL infiltration (HR: 0.59, 95%CI: 0.29–1.19) indicated opposite prognostic outcomes. This contradiction may be attributed to the complex interactions which occur in the tumor immune microenvironment. AURKA^high^ was linked to a high degree of TIL infiltration; however, the expression of various immunosuppressive molecules, such as programmed cell death-1 (PD-1) may be upregulated, which could inhibit the antitumor effects of immune cells to exert antitumor effects (46). AURKA^high^ was also found to be associated with the intratumoral localization of TILs, potentially indicating that AURKA is involved in immune regulation in MTC. In a prior study, combination treatment with the AURKA inhibitor alisertib and programmed cell death-ligand 1 (PD-L1) monoclonal antibody showed a synergistic effect in terms of tumor inhibition (47). During the combination therapy with alisertib and PD-L1 monoclonal antibody, CD8+ T cells were significantly enhanced, while the levels of interferon-gamma (IFN-γ) and tumor necrosis factor-alpha (TNF-α) in CD8+ T cells were also significantly increased (48). Therefore, we hypothesized that, compared with monotherapy with an AURKA inhibitor, combination therapy with immunosuppressants could achieve better treatment effects. However, further research is warranted to investigate this hypothesis.

This study also had some limitations which should be mentioned. Firstly, owing to the retrospective nature of the study, selection bias would have inevitably impacted the results. Secondly, patients who did not undergo surgery were not included because only patients who undergo surgery had access to complete pathological information. Thus, an inherent selection bias existed. In addition, we observed a relatively good overall prognosis of MTC in this study, with only ten disease‐related deaths). Therefore, it was not possible to evaluate the association between the degree of AURKA and mortality due to the insufficient number of events. Finally, AURKA may play an important role in the regulation of the tumor immune microenvironment; however, additional research studies are warranted to identify the potential mechanism involved in this process.

## Conclusion

AURKA expression was associated with the malignant phenotypes of MTC. The prognostic value of the combination of AURKA with TILs was found to be greater than those of either individual marker. The AURKA^high^/TILs^low^ phenotype was identified as an independent factor of BCR and STR in MTC, and could be used to accurately identify patients at high risk of incurable disease recurrence. However, the present conclusions and the actual efficacy of AURKA inhibitors should be further investigated.

## Data availability statement

The datasets presented in this study can be found in online repositories. The names of the repository/repositories and accession number(s) can be found in the article/supplementary material.

## Ethics statement

The studies involving human participants were reviewed and approved by the Ethical Committee of the Tianjin Medical University Cancer Institute and Hospital. The patients provided written informed consent to participate in this study. Human samples were collected following approval by the Ethics Committee of Tian Jin Medical University Cancer Institute and Hospital, and informed consent was obtained from all patients.

## Author contributions

ZW: Data curation, Formal analysis, Writing – original draft, Writing – review & editing. FG: Methodology, Writing – review & editing. GF: Investigation, Writing – review & editing. ZZ: Software, Writing – review & editing. NK: Validation, Writing – review & editing. XH: Supervision, Writing – original draft. XZ: Resources, Writing – original draft, Writing – review & editing.
